# P04.33. A review of existing methods to specify condition specific prevalence and experience of CAM use in US children: toward a strategic data plan

**DOI:** 10.1186/1472-6882-12-S1-P303

**Published:** 2012-06-12

**Authors:** C Bethell, S Stumbo, N Gombojav, C Wilhelm, P Newacheck

**Affiliations:** 1 The Child and Adolescent Health Measurement Initiative (CAHMI), OHSU, Portland, USA; 2 UCSF Institute for Health Policy Studies, San Francisco, USA

## Purpose

To review existing methods to specify condition specific prevalence of and experience of the use of CAM in children in the US and begin to specify a strategic data plan to support evaluation of pediatric integrative medicine.

## Methods

Qualitative (structured interviews; focus groups; concept and best practice methods mapping) and quantitative methods were used to evaluate the National Health Interview Survey’s Children’s CAM Supplement (2007), the Medical Expenditures Panel Survey (2008) and the National Survey of Children with Special Health Care Needs (2009/10), and to compare findings and specify strengths and weaknesses of each.

## Results

Using the NHIS Child CAM Supplement method for asking about reasons for the use of CAM in children, we would conclude that fewer than 15% of all children using CAM do so for specific health conditions. However, over 91.7% of all child CAM users had parents who indicated in the larger NHIS that their child had one or more of 59 health problems or conditions assessed and over half of these had 3 or more health conditions. Nearly 40% of all child CAM users met criteria for having a special health care need. Linkage of NHIS and MEPS datasets result in 2411 cases of data - insufficient to validly explore the many condition specific patterns and associations critical to better understanding the need for and impact of integrative medicine for children. NS-CSHCN findings reveal similar overall prevalence and patterns of use and offers alternative methods for consideration.

## Conclusion

NHIS Child CAM Supplement methods are not valid for assessing CAM use among children with specific health conditions. Alignment with methods used in conventional medical care will enable comparisons between and relationships among CAM and conventional medicine use in the care of children’s health conditions. A strategic approach to ensuring sufficient and valid data are collected across all national surveys is indicated.

